# Emerging Role of Long Non-Coding RNAs in the Pathobiology of Glioblastoma

**DOI:** 10.3389/fonc.2020.625884

**Published:** 2021-02-03

**Authors:** Omidvar Rezaei, Kasra Honarmand Tamizkar, Guive Sharifi, Mohammad Taheri, Soudeh Ghafouri-Fard

**Affiliations:** ^1^ Skull Base Research Center, Loghman Hakim Hospital, Shahid Beheshti University of Medical Sciences, Tehran, Iran; ^2^ Department of Medical Genetics, Shahid Beheshti University of Medical Sciences, Tehran, Iran; ^3^ Urogenital Stem Cell Research Center, Shahid Beheshti University of Medical Sciences, Tehran, Iran

**Keywords:** lncRNA, circRNA, glioblastoma, expression, polymorphism

## Abstract

Glioblastoma is the utmost aggressive diffuse kind of glioma which is originated from astrocytes, neural stem cells or progenitors. This malignant tumor has a poor survival rate. A number of genetic aberrations and somatic mutations have been associated with this kind of cancer. In recent times, the impact of long non-coding RNAs (lncRNAs) in glioblastoma has been underscored by several investigations. Up-regulation of a number of oncogenic lncRNAs such as H19, MALAT1, SNHGs, MIAT, UCA, HIF1A-AS2 and XIST in addition to down-regulation of other tumor suppressor lncRNAs namely GAS5, RNCR3 and NBAT1 indicate the role of these lncRNAs in the pathogenesis of glioblastoma. Several *in vitro* and a number of *in vivo* studies have demonstrated the contribution of these transcripts in the regulation of cell proliferation and apoptosis, cell survival, invasion and metastasis of glioblastoma cells. Moreover, some lncRNAs such as SBF2-AS1 are involved in conferring resistance to temozolomide. Finally, few circularRNAs have been identified that influence the evolution of glioblastoma. In this paper, we discuss the impacts of lncRNAs in the pathogenesis of glioblastoma, their applications as markers and their implications in the therapeutic responses in this kind of cancer.

## Introduction

Being considered as grade IV glioma tumors, glioblastomas are the utmost aggressive diffuse kind of glioma originating from the astrocytes, neural stem cells or progenitors (1). This type of brain tumor includes about half of all glioma tumors and less than 20% of all primary brain tumors (2). Although being a rare tumor, the poor prognosis and low survival rate of glioblastoma have made it an important public health problem (3). It is more frequent in men compared with females, in Western countries compared with developing world and in some ethnicities such as Asians, Latinos and Whites (3). The etiology of this kind of tumor is largely unclarified, as no causal carcinogen has been linked with it. High dose ionizing radiation is the solitary environmental element that is highly associated with risk of glioblastoma (4). A number of genetic aberrations such as activation of growth factor cascade through amplification and mutations in receptor tyrosine kinase genes, induction of the PI3K proteins and loss of the p53 and Rb tumor suppressor genes have been identified in glioblastoma (5). Genome-wide and direct sequencing techniques have also detected recurrent disease-causing mutations in glioblastoma samples in a number of genes such as *IDH1* (6) and *TERT* promoter (7). Moreover, contemporary studies have conveyed anomalous expression of long non-coding RNAs (lncRNAs) in glioblastoma samples indicating the impact of these transcripts in the pathobiology of this kind of cancer (8). These transcripts are larger than 200 nucleotides and regulate expression of numerous genes at transcriptional, post-transcriptional, and epigenetic phases (9). In the current paper, we discuss the impact of lncRNAs in the pathobiology of glioblastoma and their effects on the regulation of cell proliferation and apoptosis, cell survival, invasion and metastatic aptitude of glioblastoma cells.

## Oncogenic lncRNAs in Glioblastoma

**Figure 1:**
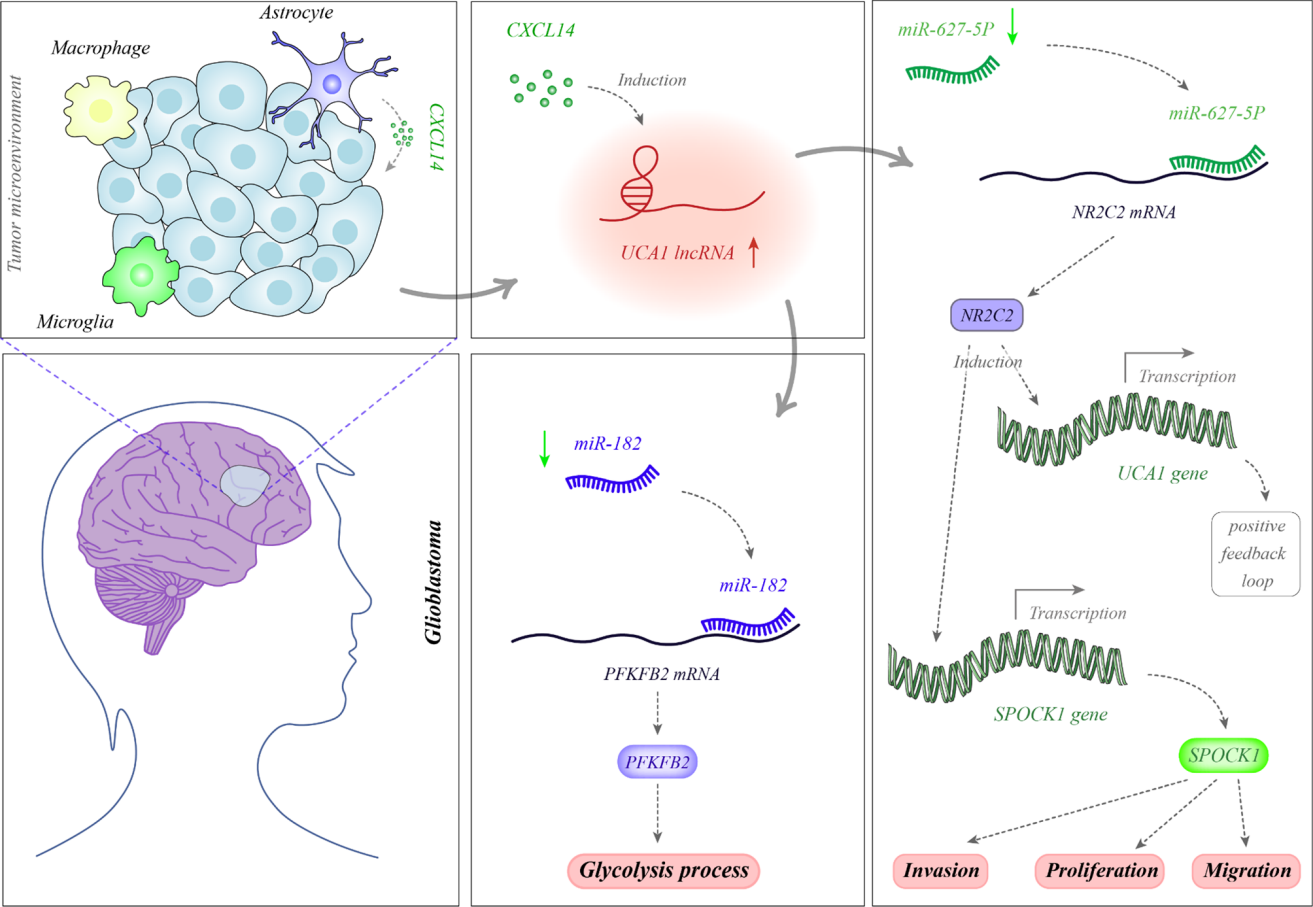
Glioblastoma-associated stromal cells (GASCs) are special cells in the tumor microenvironment which are phenotypically and functionally similar to the cancer-associated fibroblasts. These cells produce CXCL14 which functions as a paracrine factor to enhance expression of UCA1. UCA1 serves as a sponge for miR-182. Since miR-182 suppresses expression of PFKFB2, UCA1 up-regulation results in up-regulation of PFKFB2 through sequestering miR-182. PFKFB2 protein increases glycolysis in the tumor cells (16). In addition, UCA1 decreases miR-627-5p levels. As miR-627-5p inhibits NR2C2 expression, down-regulation of miR-627-5p by UCA1 enhances expression of NR2C2. NR2C2 binds with the promoter region of UCA1 and increase its expression through a positive feedback loop. Moreover, NR2C2 enhances expression of SPOCK1 increasing cell proliferation, migration and invasiveness of tumor cells (17).

Several oncogenic lncRNAs have been up-regulated in glioblastoma samples. For instance, MIR22HG is an oncogenic lncRNA which has been shown to be highly dysregulated in glioblastoma *via* assessment of accessible datasets. This lncRNA hosts miR-22-3p and miR-22-5p. Further studies have unraveled over-expression of the MIR22HG/miR-22 route in glioblastoma and glioma stem-like cells. Over-expression of MIR22HG in glioblastoma samples has been related with poor patients’ outcome. Knock down of this lncRNA has led to inactivation of the Wnt/β-catenin route *via* modulating miR-22-3p and miR-22-5p expressions. Functionally, MIR22HG silencing has diminished cell proliferation, invasion and tumor growth in xenograft models. The mentioned miRNAs have been shown to target SFRP2 and PCDH15. Taken together, MIR22HG has been acknowledged as an important activator of the Wnt/β-catenin signaling pathway, and its silencing has been proposed as a therapeutic modality in this kind of cancer (10). The small nucleolar RNA host gene 5 (SNHG5) is another up-regulated lncRNA in glioblastoma which enhances cell proliferation and suppresses cell apoptosis in these cells. Expression of this lncRNA is activated by the Yin Yang 1 (YY1) transcription factor. This lncRNA exerts its oncogenic role *via* stimulation of the p38/MAPK axis (11). SNHG9 has also been demonstrated to be over-expressed in glioblastoma samples in association with poor survival of patients. SNHG9 has a role in suppression of miR-199a-5p expression and enhancement of Wnt2 expression in glioblastoma cells. This lncRNA has been revealed to enhance aerobic glycolysis and cell proliferation (12). Expression of SAMMSON has been increased in the plasma of patients with glioblastoma but not in those with diffuse neurosarcoidosis, a disorder that shares MRI signs with glioblastoma. This lncRNA has been displayed to suppress expression of miR-622 in glioblastoma cells and subsequently enhance cell (13). MIAT is another up-regulated lncRNA in glioblastoma. Bountali et al. have knocked down this lncRNA in glioblastoma cell lines and analyzed RNA profile of these cells *via* RNA sequencing method. They reported differential expression of several genes including those participating in cancer-associated functions, namely cell growth and viability, apoptotic features, reactive oxygen species creation and migration. Functionally, MIAT silencing abolishes long-term viability and migration and enhances apoptosis in these cells (14). A genome-wide expression profiling in glioblastoma cells has identified MALAT1 as one of the most remarkably over-expressed genes following treatment with temozolomide (TMZ). Expression of this lncRNA has been co-regulated by p50 and p53 through κB- and p53-binding sites which are located in coding sequence of this lncRNA. MALAT1 silencing has increased sensitivity of patient-originated glioblastoma cells to TMZ and improved the effects of this drug in xenograft mice models (15). UCA1 is another oncogenic lncRNA which enhances cell proliferation and migration, while suppressing cell apoptosis. **Figure 1** depicts the molecular mechanisms through which UCA1 participates in the pathogenesis of glioblastoma.

**Table 1** reviews the function of oncogenic lncRNAs in glioblastoma.

**Table 1:** List of over-expressed lncRNAs in glioblastoma.

lncRNA	Patients’ specimens	Cell line	Targets/Regulators	Signaling pathways	Functional impact	Impact of high expression on patient’s prognosis	Reference
MIR22HG	18 gliomas and 5 NBT	U87MG, LN229, and LN18	b-catenin, miR-22-3p, SFRP2, PCDH15	Wnt	MIR22HG is over-expressed in glioma and glioma stem-like cells. Its silencing constrains the Wnt/b-catenin axis *via* loss of miR-22-3p and -5p. This diminishes proliferation, invasion and tumor growth.	poor survival	(10)
SNHG5	–	U251, U87, LN229 and HEB	ELK1, caspase-3, STAT1, p-p38/YY1, TNF-a	p38/MAPK	SNHG5 enhances GBM proliferation and suppresses apoptosis in GBM. YY1 is the activator of SNHG5 transcription in GBM.	–	(11)
SNHG9	–	U87 and U251	miR-199a-5p and Wnt2	Wnt/b-catenin	SNHG9 enhances aerobic glycolysis and cell proliferation, which can be weakened by miR-199a-5p.	lower survival rate	(12)
SAMMSON	56 patients with GBM, 34 patients with diffuse neurosarcoidosis and 35 healthy controls	U87, U-373	miR-622	–	SAMMSON overexpression down-regulates miR-622 and increases proliferation rate.	–	(13)
DLEU1	10 GBM tissues and 10 adjacent NBT	SHG-44, U251	TRA F4	–	Over-expression of DLEU enhances viability and cell proliferation.	–	(18)
TRG-AS1	51 glioma tissues and 51 NBTs	U251, U87, A172, LN229, NHAs	miR-877-5p	–	TRG-AS1 inhibits miR-877-5p while miR-877-5p inhibits SUZ12 expression.	poor prognosis	(19)
LINC01579	51 patients with GBM	U251, U87, U87MG, LN229, NHA	miR‐139‐5p	–	LINC01579 regulates cell proliferation and apoptosis through binding with miR‐139‐5p.	–	(20)
AGAP2-AS1	58 GBM patients	A172, U87/MG, U251/MG, LN229, SHG44, NHA	EZH2 and LSD1	–	Up-regulation of AGAP2-AS1 enhances cell proliferation and apoptosis.	Poor prognosis	(21)
lnc-TALC	79 GBM patients	LN229, U251, 551W, HG7, 229R, 251R, 551WR, HG7R	miR-20b-3p/phosphorylated AKT/FOXO3 axis	c-MET	lnc-TALC is associated with TMZ resistance through interacting with miR-20b-3p to enhance c-Met expression.	Low lnc-TALC expression improved prognosis after receiving TMZ.	(22)
LncSBF2-AS1	20 primary and their corresponding recurrent GBM specimens (each pair from the same patient who was under TMZ treatment)	U87, LN229, A172, T98, U251, HEK293T, N3 primary culture cell	miR-151a-3p/ZEB1	–	SBF2-AS1 is up-regulated in TMZ-resistant GBM cells and tissues.	Recurrent GBM patients cases with high serum exosomal SBF2-AS1 amounts had poor outcome and a resistance to TMZ.	(23)
SNHG20	78 pairs of human glioblastoma tissues and adjacent tissues	U87MG, U343, U251, LN215, NHA	Cyclin D1, CDK4, caspase 9, PI3K, Akt and mTOR	PI3K/Akt/mTOR	SNHG20 overexpression enhanced cell proliferation, decreased apoptosis and increased stem properties.	Low survival rate	(24)
MALAT1	Patients expressing MALAt1 were separated into two low (n=19) and high (n=15) expressing groups	U87, A172, and U251, patient-derived GSCs, GBM34 and GBM44	-/p50 and p52	NF-κB	MALAT1 silencing sensitizes GBM cells to TMZ.	–	(15)
	–	U251, U87	ZEB1, MDR1, MRP5, LRP1	–	MALAT1silencing down-regulated MDR1, MRP5, and LRP1 levels, increased response to TMZ, and decreased ZEB1 level.	–	(25)
	–	U251	miR-101, GSK3β, MGMT	–	MALAT1 levels were higher in TMZ-resistant GBM cells. MALAT1 silencing reduces TMZ resistance by inhibiting cell proliferation and promoting apoptosis.	–	(26)
	140 GBM patients: 70 responsive to TMZ and 70 non-responsive	U87, U251	miR-203, TS	–	MALAT1 induces resistance to TMZ *via* inhibiting miR-203 and enhancing thymidylate synthase expression.	Poor OS and RFS	(27)
miR155HG	24 GBM tissues and 15 adjacent NBTs	normal human astrocyte cell line NHA, U87, U251, Ln229, T98, and A172, GP1 and GP2	miR-185/ANXA2, STAT3	PI3K-Akt	miR155HG enhances epithelial-to-mesenchymal transition in glioma. miR155HG silencing inhibited GBM cell proliferation, stimulated G1/S-phase cell cycle arrest, and enhanced apoptosis.	–	(28)
TP73-AS1	TCGA and GTEx datasets: 207 normal, 518 low grade glioma and 163 GBM	G26 and G7	ALDH1A1	–	TP73-AS1 increases TMZ resistance in GBM cancer stem cells and enhances tumor aggressiveness.	Poor prognosis	(29)
LINC-ROR	57 GBM tissues and 10 NBTs	–	caspase 3 and p53	–	Patients with OS less than 15 months had up-regulation of LINC-ROR.	poor PDF and overall survival	(30)
MIAT	–	SH-SY5Y, GBM 1321N1, GBMT98G	Many genes	MAPK, Phospholipase D, TGF-β, NOD-like receptor, EGFR	MIAT enhances cell growth, survival, production of reactive oxygen species and migration, and decreases basal apoptosis.	–	(14)
HOXB-AS1	486 low grade glioma (LGG) and 154 glioblastoma (GBM) tissues	HA, LN229, U87 and U251 cell lines	miR-885-3p, HOXB2		HOXB-AS1silencing suppresses cell proliferation through inducing S phase cell cycle arrest, and suppresses the migration and invasion capacity.	Poor prognosis	(31)
GAPLINC	High GAPLINC expressing group (n=80) and low GAPLINC expressing group (n=81)	NHAs,T98G, U251, LN18, LN229, and A172	miR-331-3p	–	GAPLINC enhances GBM cells proliferation, migration, and invasion, and reduces apoptosis.	shorter overall survival and disease-free survival	(32)
AHIF	–	U87-MG and T98G GBM cell	Bax, Bcl-2, and caspase 7	–	AHIF was up-regulated in GBM cells after radiotherapy and affects GBM cell clonogenic formation, DNA repair and apoptosis.	–	(33)
	31 GBM patients and 7 adjacent NBT	U87-MG, U251-MG, A172, T98G	VEGF, angiogenin, Bcl-2, Bcl-xl, Mcl-1	–	AHIF enhances viability and invasiveness, and reduced the proportion of apoptotic cells. Exosomes originated from AHIF−overexpressing GBM cells enhanced viability, invasion and radio-resistance.	–	(34)
AGAP2‐AS1	116 GBM tissues, 20 low‐grade glioma samples and 20 adjacent NBTs	U87, U251, human astrocyte cell line (HA)	–	–	Up-regulation of AGAP2‐AS1 enhances cell proliferation, migration, and invasion, but reduces cell apoptosis.	Shorter overall survival	(35)
lnc-UCA1	Glioma samples: Grade I–II (n=5), Grade III–IV (n=5) and normal human brain tissues (n=5)	Human U87 and U251 glioma cell	miR-627-5p, NR2C2	–	UCA1 overexpression enhances proliferation, migration, and invasion, but suppresses apoptosis.	–	(17)
	42 paired glioma tissues and NBTs	U251, U87MG	miR-182, PFKFB2/CXCL14	–	UCA1/miR-182/PFKFB2 axis induces glycolysis and invasion.	Poor survival	(16)
H19	50 FFPE brain tissue from GBM patients and 10 cancer-free brain tissue samples	–	miR-326	–	H19 over-expression confers poor OS and progression-free survival.	Poor OS	(36)
	–	U87, U251, Ln229, U373, U118, GP1, GP2	miR-181d, β-catenin/Hif-1α, PTEN, SP1	–	H19 expression is increased by Hif-1α under hypoxia. H19 contributes in hypoxia-associated migration and invasion.	Lower survival rate	(37)
	30 glioblastoma tissues and adjacent NBT	U87, U373, HUVECs	–	–	H19 enhances glioblastoma cell invasion, neurosphere formation, tumor growth and angiogenesis.	Lower PFS	(38)
	–	U87MG, U251, U343, Hs683, LN215, A172, NHA	–	–	H19 silencing reduced cellular proliferation and increased apoptosis rate when induced by TMZ. Cancer stem cell markers (CD133, Nanog, Oct-4, and Sox2) are increased by H19 upregulation.	–	(39)
LINC00152	35 samples (5 normal, 10 with grade two, 9 with grade three and 11 with grade four GBM)	LN229, U87-MG and N9 (patient-derived cells)	miR-612	AKT2/NF-κB	LINC00152 regulates malignant progression and proneural–mesenchymal transition.	Poor prognosis	(40)
	–	U87	TPM2, PTX3, IGFBP4, TGM2, SPP1, LUM	–	LINC00152 increases cellular invasion and EMT.	Poor survival	(41)
	40 glioblastoma samples and matched NBTs	U87, U251, LN229, A172, U118, NHA	E-cadherin, N-cadherin, Vimentin, and Snail, HMGA2	–	LINC00152 enhances cell proliferation, EMT and invasion.	–	(42)
LINC00470	50 GBM samples and 10 NBTs	U251, U87 and U118	ELFN2, miR-101, AurkA. and eIF2a		LINC00470 increases expression of ELFN2 and regulates methylation of ELFN2. LINC00470 suppresses ELFN2-induced GBM cell autophagy.	Poor prognosis	(43)
	60 astrocytoma tissues and 12 NBT	U251, U87	FUS and AKT	–	Higher pAKT induced by LINC00470 decreased ubiquitination of HK1 and suppressed autophagy. Higher LINC00470 expression was associated poor patient outcome.	Poor prognosis	(44)
LINC01446	31 pairs of GBM samples and adjacent normal tissues	NHA, A172, U87, U251 and T98G	miR-489-3p, TPT1		LINC01446 silencing suppressed GBM cell proliferation, arrested cell-cycle progression, decreased tumor growth and attenuated invasion.	poor prognosis and OS	(45)
CASP5	40 pairs of GBM and NBTs	A172, U87MG, U251MG, T98G, U118MG and the human astrocyte cell line HA	Cyclin D1, MMP-9, MMP-2, E-cadherin, N-cadherin, and Vimentin	–	CASP5 silencing has suppressed GBM proliferation and arrested cells in G1.	–	(46)
LOXL1-AS1	169 GBM RNA-seq data (68 MES and 101 PN)	U87MG	RELB	NF-kB	GBM cell proliferation was inhibited by LOXL1-AS1 silencing.	Poor prognosis and low OS	(47)
MNX1-AS1	44 pairs of GBM samples and adjacent normal tissues	U138, LN229, T98, U251	miR-4443	–	MNX1-AS1 enhanced the proliferation, migration, and invasion of GBM cells.	–	(48)
HOTAIR	43 GBM patients and 40 controls	–	–	–	HOTAIR expression correlates with high grade brain tumors.	–	(49)
	123 GBM cases from TCGA, 34 cases from CGGA2, 227 cases from Rembrandt, 79 cases from TTseq, and 77 cases from GSE4290	U87, U87vIII	NLK	β-catenin	HOTAIR silencing suppressed GBM cell migration and invasion.	Poorer survival	(50)
	TCGA dataset: 220 glioma	U87, LN229	EZH2	–	HOTAIR enhances cell cycle progression.	Lower survival	(51)
SNHG7	53 pairs of GBM tissues and NBTs	HEB, A172, U87, T98G, SHG44	miR-5095	Wnt/b-catenin	SNHG7 silencing inhibited proliferation, migration and invasion and induced apoptosis.	Poorer prognosis	(52)
NEAT1	–	N5, N9 and N33 patient-derived cells	b-catenin, ICAT, GSK3B, Axin2, EZH2/STAT3,p65	WNT/b-catenin, EGFR, NFkB	NEAT1 enhances proliferation, clone formation, and invasion but suppresses cell apoptosis.	–	(53)
	120 glioma tissues and 30 NBTs	U87, T98G, U251, A272, U373, HEK293T	miR-let7e, Argonaute 2, NRAS	–	NEAT1 silencing suppressed GSC cell proliferation, migration and invasion and promoted GSC apoptosis.	–	(54)
SOX2OT	Human glioma tissues (grade one=5, grade two=5, grade three=8, grade four=8) and 5 NBTs	U87 and U251	miR-194-5p and miR-122/SOX3	JAK/STAT	SOX2OT promoted the proliferation, migration and invasion of GSCs, and inhibited GSCs apoptosis.	–	(55)
TUG1	20 GBM specimens (grade one to four, each 5) and 5 normal brain tissues	U251 MG, U87MG, 293T	miR-299, VEGFA	–	TUG1 promotes tumor-induced endothelial cell proliferation, migration and tube formation and enhances spheroid-based angiogenesis.	–	(56)
HIF1A-AS2	–	Primary human GSCs	IGF2BP2, DHX9, HMGA1	–	This lncRNA regulates GSC growth, self-renewal, hypoxia-associated molecular reprogramming and adaptation to hypoxia within the tumor niche.	Poor OS	(57)
XIST	–	Human embryonic kidney (HEK) 293T cells	miR-152	–	XIST promotes cell proliferation, migration and invasion and suppresses apoptosis.	–	(58)
MCM3AP-AS	422 GBM patients (TCGA dataset)	–	MCM3AP	–	MCM3AP-AS corresponds to the coding-gene MCM3AP, which is involved in initiation of DNA replication.	Lower OS	(59)
LINC01057	12 paired frozen fresh GBM and adjacent NBTs and the paraffin-embedded human GBM samples	LN229, T98G, HEK293T	IKKα	NF-κB	LINC01057 up-regulation increases mesenchymal differentiation in proneural cells.	–	(60)

GBM, glioblastoma multiform; TMZ, temozolomide; OS, overall survival; GSC, glioblastoma stem cell; NBT, normal brain tissues.

## Tumor Suppressor lncRNAs In Glioblastoma

Expression of GAS5 has been decreased in glioblastoma and its levels have been negatively correlated with miR-34a levels (61). In addition, expression of AC016405.3 has been decreased in glioblastoma tissues in association with numerous aggressive characteristics of this type of cancer. Up-regulation of this lncRNA inhibits proliferation and metastatic ability of glioblastoma cells. The oncogenic miRNA, miR-19a-5p has been identified as a downstream miRNA of AC016405.3. AC016405.3 has been shown to be targeted by miR-19a-5p. Functionally, AC016405.3 inhibits cell proliferation and metastasis *via* regulation of TET2 by serving as a sponge for miR-19a-5p (62). LINC00657 is another tumor suppressor lncRNA whose expression has been decreased in glioblastoma sections compared with neighboring normal section. Up-regulation of this lncRNA has suppressed cell proliferation, colony formation, invasiveness and migratory potential of glioma cells through activating cell apoptosis. LINC00657 has been acknowledged as a direct target of miR-190a-3p, a miRNA that negatively regulates PTEN expression. The tumor suppressive role of LINC00657 has also been verified in xenograft models (63). The lncRNA AC003092.1 has been shown to be down-regulated in TMZ resistance cells compared with their original cells. Moreover, down-regulation of this lncRNA has been correlated with resistance to TMZ, higher possibility of tumor relapse, and poor patients’ outcome. Cell line studies has shown improvement of TMZ sensitivity following up-regulation of AC003092.1. The effect of this lncRNA in the modulation of TMZ sensitivity is exerted *via* regulation of TFPI-2–associated cell apoptosis through sponging miR-195 (64). RNCR3 is another down-regulated lncRNA in glioblastoma. Over-expression of this lncRNA significantly suppresses cell survival and proliferation of glioblastoma cells, while enhancing cell apoptosis and activity caspase‐3/7. Besides, up-regulation of this lncRNA enhances expression of Krüppel‐like factor 16 (KLF16) *via* suppressing miR‐185‐5p (65). **Table 2** gives an outline of studies which assessed function of tumor suppressor lncRNAs in glioblastoma.

**Table 2:** List of under-expressed lncRNAs in glioblastoma.

lncRNA	Patients’ specimens	Cell line	Targets/Regulators	Signaling pathways	Functional role	Impact of low expression on patient’s prognosis	Reference
AC016405.3	3 GBM samples and paired NBTs, 64 FFPE GBM specimens	U87MG, U251MG	miR-19a-5p, TET2	–	AC016405.3 inhibits proliferation and metastasis *via* affecting expression of TET2.	Poor prognosis	(62)
LINC00657	40 pairs of GBM tissues and adjacent normal tissues	HA1800, U-87, LN-18, and U-118 MG	miR-190a-3p	pTEN	LINC00657 suppresses viability and colony formation in through increasing cell apoptosis.	Poor progonosis	(63)
AC003092.1	108 human glioma tissue samples (75 grade IV, 5 grade III, 13 grade II, and 15 grade I astrocytoma cases)	U87, U251 and their TMZ-resistant lines, U87TR and U251TR	TFPI-2, miR-195	–	Down-regulation of AC003092.1 correlates with TMZ resistance, higher risk of relapse, and poor outcome.	Poor prognosis	(64)
GAS5	50 FFPE GB specimens and 10 NBTs	–	miR-34a	–	GAS5 level in reduced in GBM.	Poor overall survival	(61)
RNCR3	–	U87, U251, U373, A172	miR‐185‐5p, KLF16	–	RNCR3 overexpression suppresses cell survival and proliferation, enhances cell apoptosis and activity of caspase‐3/7.	–	(65)
NBAT1	48 cases of GBM (two groups of low=24 and high=24 expression of NBAT1) and 30 cases of normal brain tissues	SVGP12, U251, U87, U373, T98, and LZ229	Akt	–	NBAT1 down-regulation correlates with proliferation ability, tumor size, degree of malignancy and cell viability.	Lower OS and poor prognosis	(66)
TUSC7	116 GBM specimens, 72 insensitive and 44 sensitive to TMZ treatment	U87	miR-10a	–	Under-expression of TUSC7 confers resistant to TMZ.	–	(67)
RAMP2-AS1	20 GBM patients and adjacent normal tissue	U87 and U251	NOTCH3, P21, DHC10	NOTCH	RAMP2-AS1 suppresses GBM cell growth and enhances cell cycle progression.	Poor prognosis	(68)
RP11-838N2.4	53 patients: 38 GBM cases, 3 grade III astrocytoma cases, 10 grade II astrocytoma cases, 2 grade I astrocytoma cases	U87TR, U251TR, U87, U251	miR-10a, EphA8	TGF-β	Down-regulation of RP11-838N2.4 was correlated with higher probability of tumor relapse.	Poorer survival	(69)

GBM, glioblastoma multiform; TMZ, temozolomide; OS, overall survival; GSC, glioblastoma stem cell; FFPE, formalin-fixed, paraffin paraffin-embedded.

## Diagnostic and Prognostic Value of lncRNAs in Glioblastoma

Expression levels of lncRNAs can distinguish patients with glioblastoma from cancer-free individuals. Moreover, these transcripts can possibly differentiate different brain tumors. For instance, plasma levels of SAMMSON can differentiate glioblastoma from both diffuse neurosarcoidosis and healthy controls (13). Among lncRNAs whose diagnostic power has been assessed in glioblastoma, HOTAIR has exhibited the most promising results. Tan et al. have demonstrated significant higher levels of this lncRNA in sera of glioblastoma patients compared with controls. The area under the receiver operating characteristic (ROC) curve was 0.913 indicating the ideal feature of HOTAIR for this purpose. Moreover, they reported significant correlation between its levels and high tumor grade. Notably, there was significant correlation between tumor and serum levels of this lncRNA. Finally, exosomes extracted from the serum samples have been shown to contain this lncRNA, further emphasizing the application of this lncRNA in the prognostic and diagnostic processes in glioblastoma (49). In addition, Kaplan-Meier analysis has indicated the correlation between expression levels of several lncRNAs such as SNHG9, TRG-AS1, AGAP2-AS1, lnc-TALC, SBF2-AS1, SNHG20, AC016405.3, LINC-ROR, HOXB-AS1, H19, LINC00152, RAMP2-AS1 and GAS5 and patients’ prognosis in the terms of overall survival, disease-free survival and progression free survival. **Table 3** gives a summary of studies which assessed such aspect of lncRNAs in glioblastoma.

**Table 3:** Diagnostic/prognostic value of lncRNAs in glioblastoma.

Sample number	Area under curve	Sensitivity	Specificity	Kaplan–Meier analysis	Univariate/Multivariate Cox regression	Reference
Two groups of high and low SNHG9 expressing patients, each contained 20 patients	–	–	–	OS and PFS in patients with high SNHG9 expression were lower than those with down-regulation of SNHG9. High SNHG9 expression was correlated with high tumor grade, greater tumor dimension, and metastasis.	SNHG9 was an independent prognostic factor for worse OS.	(12)
56 patients with GBM, 34 patients with diffuse neurosarcoidosis and 35 healthy controls/SAMMSON levels	GBM versus diffuse neurosarcoidosis: 0.92 GBM versus healthy controls: 0.88	–	–	–	–	(13)
51 samples of glioma tissues	–	–	–	TRG-AS1 has been related with poor prognosis.	–	(19)
58 GBM patients	–	–	–	Higher levels of AGAP2-AS1 correlated with lower OS.	–	(21)
79 GBM patients	–	–	–	OS in patients with TMZ therapy and low expression of lnc-TALC was increased, whereas high expression of lnc-TALC and therapy with TMZ reduced OS.	TMZ chemotherapy was correlated with the OS of patients with low lnc-TALC expression.	(22)
77 with high levels of SBF2-AS1 and 77 with low levels of SBF2-AS1	–	–	–	OS decreases in patients with high levels of SBF2-AS1.	–	(23)
45 patients with low levels of SNHG20 and 33 patients with high levels of SNHG20	–	–	–	High levels of SNHG20 was correlated with lower rate of OS.	–	(24)
Two groups of 32 patients with high and low levels of AC016405.3	–	–	–	Low expression of AC016405.3 was correlated with a shorter survival rate, a larger size of tumor, a higher grade, and more common distant metastasis.	–	(62)
57 glioblastoma patients	0.653 ± 0.078	65.4	77.8	Patients with high LINC-ROR amounts had poor survival.	–	(30)
LGG (n=486) and GMB (n=154)	–	–	–	High expression of HOXB-AS1 was associated with poorer prognosis in GBM.	–	(31)
136 glioma patients	–	–	–	High levels of AGAP2-AS1was correlated with lower OS.	–	(35)
high (n = 37) and low (n = 38) AC003092.1 expression group	–	–	–	High AC003092.1 expression group indicated higher OS.	–	(64)
50 FFPE brain tissue from GBM patients	0.686 (0.537–0.836)	71.4	59.6	H19 overexpression correlates with poorer OS.	–	(36)
CGGA GBM (high expression= 45 and low expression= 45), TCGA GBM (high expression = 77 and low expression= 78)	–	–	–	Higher expression of LINC00152 correlates with lower OS.	LINC00152 levels, age, chemotherapy and radiotherapy have been associated with OS in CGGA database. LINC00152 levels, age, IDH status, and chemotherapy have been associated with OS database.	(40)
Low group (n=15) and high group (n=16)	–	–	–	Patients with a higher LINC01446 expression had a poor survival rate in five years.	–	(45)
15 patients with GBM/HOTAIR	0.913	86.1	87.5	–	–	(49)
53 patients with GBM	–	–	–	Higher expression of SNHG7 correlated with poorer survival rate.	–	(52)
20 patients with GBM	–	–	–	Lower survival rate with lower expression of RAMP2-AS1.	–	(68)
53 patients: 38 GBM cases, 3 grade III astrocytoma cases, 10 grade II astrocytoma cases, 2 grade I astrocytoma cases	–	–	–	High level of lncRNA RP11-838N2.4 has been correlated with longer survival.	–	(69)
LINC00470 expression levels in two groups: high=37, low=38	–	–	–	High LINC00470 amounts were correlated with shorter survival times and poor prognosis.	LINC00470 levels, astrocytoma grade, age, and tumor site were associated with OS.	(44)
7 low HOTAIR and 26 high HOTAIR (for survival), 22 low HOTAIR and 46 high HOTAIR (for DFS), 10 high GAS5 and 23 low GAS5 (for survival), 21 high GAS5 and 47 low GAS5 (for DFS)	–	–	–	Patients with high HOTAIR and low GAS5 levels had worse survival rates relative to patients with low HOTAIR and high GAS5 levels.	–	(70)
Low (54) and high (54) groups of HOTAIR expression (CGGA1 dataset)	–	–	–	Low HOTAIR expression has increased OS.	HOTAIR over-expression, age at diagnosis, IDH1 mutation, KPS score, and Ki-67 expression were associated with OS.	(50)
Expression of H19 in two groups: high=14, low=16	–	–	–	H19 over-expression was significantly associated with a poor PFS.	–	(38)
70 high and 70 low patients of MALAT1 expression	0.775	71.51	62.82	MALAT1 over-expression was correlated with poor OS and RFS.	Serum MALAT1 levels and tumor grade were independent prognostic factors for OS of patients receiving TMZ.	(27)

GB, glioblastoma multiform; TMZ, temozolomide; OS, overall survival; PFS, progression-free survival; RFS, recurrence-free survival; DFS, disease-free survival.

## Circular RNAs and Glioblastoma

In addition to lncRNAs, Circular RNAs (circRNAs) can act as miRNA sponges to modulate expression of their target genes. Numerous studies have assessed expression and function of circRNAs in glioblastoma. For instance, Wang et al. have reported over-expression of some circRNAs and lncRNAs in miR-422a–downregulated glioblastoma samples. They have also recognized a new circRNA originated from NT5E, termed circNT5E. Expression of this circRNA is modulated by ADARB2 through binding to sites neighboring circRNA-creating introns. circNT5E has been shown to regulate cell proliferation, migration, and invasion of glioblastoma cells through binding with miR-422a and suppressing its activity (71). Li et al. have demonstrated down-regulation of circ_0001946 and *CDR1*, while up-regulation of miR‐671‐5p in glioblastoma cells. Circ_0001946 has been shown to inhibit expression of miR‐671‐5p, therefore enhancing *CDR1 *expression. Circ_0001946 and *CDR1 *decrease cell proliferation, migration, and invasion and induce apoptosis in glioblastoma cells as verified by both *in vitro* and *in vivo* assays (72). **Table 4** summarizes the expression and function of circRNAs in glioblastoma.

**Table 4:** List of circRNAs which participate in the development of glioblastoma.

circRNA	Pattern of expression	Patients’ specimens	Cell line	Targets/Regulators	Signalingpathways	Function	Patient’sprognosis	Reference
circNT5E	↑	39 pairs of glioma and NBTs	U87, U251	miR-422a/ADARB2	–	circNT5E suppresses activity of miRNAs with tumor-suppressor like features, and increase several pathologic processes, such as cell proliferation, migration, and invasion.	–	(71)
circ_0001946	↓	–	U87, U251	miR‐671‐5p, CDR1	–	Circ_0001946 inhibits expression of miR‐671‐5p, and increases CDR1 levels. Circ_0001946 and CDR1 decrease proliferation, migration, and invasion and upsurge apoptosis.	–	(73)
circMTO1	↓	59 pairs of GBM and NBTs	NHA, A172, U251, U87, SNB19, SHG44	WWOX, miR-92	–	circMTO1 suppresses proliferation of tumorous cells. circMTO1 increases expression of WWOX, and WWOX mediates circMTO1-associated suppression of proliferation of U251 cells. circMTO1 directly interact with miR-92.	Lower OS	(74)
circ-PITX1	↑	58 pairs of GBM and NBTs	A172, LN229, U251, U87, NHA	miR-379–5p, MAP3K2	MAPK	Down-regulation of circ-PITX1 inhibits cell proliferation and enhances cell apoptosis.	–	(75)
hsa_circ_0076248	↑	–	U251, U87, HEB	miR‐181a, SIRT1, p53	–	hsa_circ_0076248 sponges miR‐181a and down-regulates it. Down-regulation of hsa_circ_0076248 depresses the proliferation and invasion of glioma, and enhances the TMZ sensitivity.	–	(76)
circMMP9	↑	18 pairs of GBM and NBTs	U87, U251	miR-124, CDK4, AURKA/eIF4A3	–	circMMP9 enhances the proliferation, migration and invasion capacities.	–	(77)
circ_0074027	↑	50 pairs of GBM and NBTs	U87, U251, A172, LN229, NHA	miR-518a-5p, IL17RD	–	Cell growth, clone formation, migration and invasion were increased by circ_0074027.	–	(78)

GBM, glioblastoma multiform; TMZ, temozolomide; OS, overall survival; NBT, normal brain tissue.

## Discussion

Both candidate gene and high throughput expression studies have reported anomalous expression of several lncRNAs in glioblastoma samples indicating the oncogenic roles for some lncRNAs and tumor suppressor roles for a number of other lncRNAs. Yet, the function of the former group of lncRNAs has been more assessed in this kind of cancer. Like other cancers, the role of lncRNAs in the pathogenesis of glioblastoma can be exerted through their effects on the expression of miRNAs. Accordingly, several lncRNA/miRNA/mRNA axes have been identified in this context among them are SNHG9/miR-199a-5p/Wnt2, MIR155HG/miR-185/ANXA2, TRG-AS1/miR-877-5p/SUZ12, LINC01579/miR‐139‐5p/EIF4G2, AC016405.3/miR-19a-5p/TET2, AC003092.1/miR-195/TFPI-2, LINC00657/miR-190a-3p/PTEN, RNCR3/miR‐185‐5p/KLF16, and MALAT1/miR-203/thymidylate synthase axes. Thus, comprehensive assessment of these three types of transcripts would facilitate identification of the molecular pathways underlying the pathogenesis of this type of cancer. Moreover, a number of recent studies revealed the role of circRNAs in regulation of expression of miRNAs, thus adding an extra level of complexity in gene regulation networks. An example of the circRNA/miRNA/mRNA functional axis in glioblastoma is represented by circ_0001946/miR‐671‐5p/*CDR1*.

Association between lncRNA expression levels and resistance to TMZ has been assessed in several studies. Notably, expressions of oncogenic lncRNAs lnc-TALC, LncSBF2-AS1, MALAT1, TP73-AS1, and H19 as well as expression of tumor suppressor lncRNAs AC003092.1, TUSC7, and RP11-838N2.4 have been shown to alter this phenotype in glioblastoma cells. Therefore, a panel of these lncRNAs might be applied to predict response of pateints to this chemotherapeutic agent and establish a personalized strategy for these patients.

Finally, several oncogenic and tumor suppressor lncRNAs have been identified as modulators of glioblastoma patients’ survival indicating the appropriateness of these transcripts as prognostic biomarkers. The diagnostic power of lncRNAs SAMMSON, HOTAIR, MALAT1, H19, and LINR-ROR has been assessed in serum or tissue samples of pateints with glioblastoma revealing the best results for the first two mentioned lncRNAs based on the high values of the area under the reciver operating characteristic curves. Considering the unavialbility of tissue samples for the purpose of early diagnosis and ambiguity of imaging techniques in early stages of the disease, assessment of expression of lncRNAs in serum samples provides a non-invasive method for early detection of this kind of malignant tumor.

In brief, dysregulation of several lncRNAs has been deteceted in glioblastoma cells leading to abnormal regualtion of cancer-associated pathways and cellular processes namely apoptosis, proliferation and survival. These transcripts provide promising tools for early detection of glioblastoma and prediction of patients’ prognosis and response to therapeutic choices particularly TMZ. However, a limitation of *in vitro* studies in this regard is that most of them has been executed using traditional serum-grown cell lines such as U87 or U251. Furhther functional *in vitro* and *in vivo* investigations are required to verify the obtained data.

## Author Contributions

MT and SG-F wrote the draft and revised it. KHT, GS, and OR performed the data collection and designed the tables. All authors contributed to the article and approved the submitted version.

## Conflict of Interest

The authors declare that the research was conducted in the absence of any commercial or financial relationships that could be construed as a potential conflict of interest.
